# Infection Responsive Smart Delivery of Antibiotics Using Recombinant Spider Silk Nanospheres

**DOI:** 10.3390/pharmaceutics13091358

**Published:** 2021-08-28

**Authors:** Pranothi Mulinti, Jacob Shreffler, Raquib Hasan, Michael Dea, Amanda E. Brooks

**Affiliations:** 1 Department of Pharmaceutical Sciences, North Dakota State University, Fargo, ND 58102, USA; pranothimulinti@gmail.com (P.M.); jacob.shreffler@ndsu.edu (J.S.); raquib.hasan@ndsu.edu (R.H.); 2 College of Osteopathic Medicine, Rocky Vista University, Ivins, UT 84734, USA; Michael.dea@rvu.edu; 3 Department of Molecular Biology, Rocky Vista University, Ivins, UT 84734, USA

**Keywords:** spider silk, infection-responsive, antibiotic resistance, septic arthritis

## Abstract

Frequent and inappropriate usage of antibiotics has changed the natural evolution of bacteria by reducing susceptibility and increasing resistance towards antibacterial agents. New resistance mechanisms evolved in the response to host defenses and pharmaceutical interventions are threatening our ability to treat common infections, resulting in increased mortality. In the face of this rising epidemic, antibiotic drug discovery, which has long been overlooked by big pharma, is reaching a critical low. Thus, the development of an infection-responsive drug delivery system, which may mitigate multidrug resistance and preserve the lifetime of our current antibiotic arsenal, has garnered the attention of both popular science and funding agencies. The present work describes the development of a thrombin-sensitive linker embedded into a recombinant spider silk copolymer to create a nanosphere drug delivery vehicle. Recent studies have suggested that there is an increase in thrombin-like activity during *Staphylococcus aureus* infection; thus, drug release from this new “smart” nanosphere can be triggered in the presence of infection. A thrombin sensitive peptide (TSP) was synthesized, and the thrombin cleavage sensitivity was determined by HPLC. The results showed no cleavage of the peptide when exposed to human serum whereas the peptide was cleaved when incubated with *S. aureus* exudate. Subsequently, the peptide was coupled with a silk copolymer via EDC-NHS chemistry and formulated into nanospheres encapsulating antibiotic vancomycin. These nanospheres were evaluated for in vitro infection-responsive drug release and antimicrobial activity. Finally, the drug responsive nanospheres were assessed for efficacy in an in vivo septic arthritis model. Our study provides evidence that the protein conjugate was enzyme responsive and can be used to formulate targeted drug release to combat infections against multidrug-resistant bacterial strains.

## 1. Introduction

Antibiotics are an essential component of infection treatment and prevention. The care of patients with serious infections both within and outside healthcare settings is increasingly complicated by the high prevalence of resistant or multidrug-resistant (MDR) pathogens. Infections caused by MDR pathogens are usually associated with higher mortality and healthcare costs [1]. Although clinical antimicrobial misuse and overuse are the primary drivers for the development of resistance, poor patient compliance plays an equally important role in promoting spontaneous mutation and horizontal gene transfer that lead to the evolution of resistance. Recent research has also confirmed that sub-lethal concentrations of antibiotics can also convey resistance [2,3,4]. Thus, the necessity to use elevated concentrations of vancomycin, traditionally thought of as an antibiotic of last resort, are now commonplace, prompting concerns that society is reverting to a pre-antibiotic era.

Extending the life of current antibiotics to address the rising tide of resistance requires a paradigm shift away from new drug and small molecule discovery and development toward better delivery systems. Irrespective of the type of resistance mechanism and the spread of resistance, combatting it requires multiple levels of intervention, including new and modified antibiotics, antibiotic conjugates, and cutting-edge drug delivery systems to address this rising global problem. Systemic administration of antibiotics has been the mainstay of antimicrobial therapy for infectious disease control for decades [5]. Unfortunately, systemic delivery suffers from several significant drawbacks (e.g., poor penetration to wound and post-operative tissues, systemic toxicity, poor patient compliance, disruption of the patient’s healthy flora, etc.) that limit it as a stable antibiotic delivery strategy. Furthermore, sub-therapeutic antibiotic concentrations are known to inadvertently promote the development and spread of resistance [6]. Regardless of the underlying cause, multidrug-resistant bacteria are quickly outpacing antibiotic discovery and development. As the current pipeline is not adequate to effectively address antibiotic resistance, alternate and better use of our current arsenal may help to mitigate the resistance trend. While many delivery strategies including topical, oral, intravenous, etc., facilitate systemic drug bioavailability, local release seeks to provide therapeutic drug concentrations only to intended target sites to produce the desired pharmacological effect. Local delivery systems are frequently considered particularly to address thrombosis, osteomyelitis, periodontitis, biomedical device-related infections, and other microbial pathologies, that are refractory to most conventional methods of systemic drug administration [6,7]. Adding to this, the development of “smart” responsive delivery systems may prove an effective strategy and provide a synergistic effect to extend the effective lifetime of current therapeutics. Targeted nanotechnology-based drug delivery systems that respond to local environments are emerging as an effective approach to (1) decrease the dose and frequency of administration to improve the therapeutic index, (2) target organ and even intracellular compartments to limit systemic side effects, and (3) mitigate resistance that results from sub-therapeutic antibiotic exposure.

Strategies utilizing microenvironments unique to tissues and disease states as a molecular cue to activate drug release has gained widespread attention in the treatment of various diseases like cancer, diabetes, and bacterial infections as it increases drug stability and therapeutic efficacy while decreasing toxic side effects at the same time [8,9,10,11]. Several enzymes including a thrombin-like enzyme in *S. aureus*, myeloperoxidase in *Candida*, and esterases in uropathogens were reported to be elevated during infection [11,12], whereas virulence factors for certain bacteria like bacterial phosphatase and phospholipase are reported in other studies [11]. These enzymes act as biomarkers to develop therapeutic targets for an infection-responsive drug release system. *S. aureus-* and *P. aeruginosa*-infected wound fluid has been reported to have high thrombin-like enzymatic cleavage activity due to the bacterial enzyme staphylocoagulase [13,14,15]. Thrombin, an endogenous human protease, cleaves fibrinogen at the Arg-Gly bonds with a unique substrate specificity and is typically found at approximately 150 mg/L in the blood. Staphylocoagulase also has a thrombin-like function, which activates prothrombin to form staphylothrombin and mediates cleavage of fibrinogen to fibrin [16]. It was found that thrombin-like activity is elevated in both *S. aurues* and *P. aeruginosa* infections due to the activity of staphylocoagulase. Thus, it can be used as a trigger to release the antibiotic in the presence of infection. Previous research has optimized the sequence of the substrate (thrombin-sensitive peptide), GF_D_PRGFPAGG, which demonstrated complete cleavage within 15 min, releasing gentamicin linked to a polyvinyl alcohol (PVA) support matrix [17].

**Figure 1:**
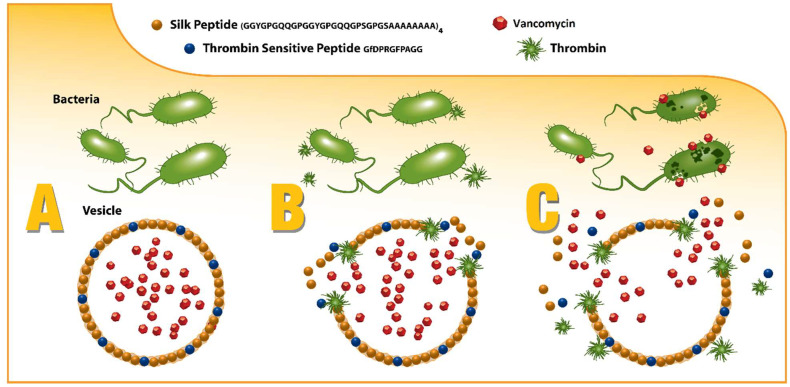
Graphical representation of the infection responsive release of the drug triggered by bacterial enzyme.

Spidroins (i.e., spider silk proteins) are a natural, structural, block copolymer, with a demonstrated combination of robust mechanical properties, biocompatibility, and a well-established structure–activity relationship, which can be easily customized according to rates of degradation to non-toxic byproducts in vivo, making them a versatile biomaterial to be increasingly considered for various biomedical and drug delivery applications [18,19,20]. By capitalizing on this “plug-and-play” motif structure, spidroins can be directly engineered to not only incorporate the thrombin-sensitive peptide element, but also to promote self-assembly into drug-encapsulating micelles and microcapsules that can release their drug payload only in the presence of infection as depicted in Figure 1 [21,22,23,24].

## 2. Methods

### 2.1. Protein Expression and Purification

The cloned consensus sequence of MaSp2 (GGYGPGQQGPGGYGPGQQGPSGPGSAAAAAAAA)_2_ was transformed into *E. coli* BL21(DE3) pLysS cells (Promega, Madison, WI, USA) for the expression of silk protein (sx2). Briefly, cultures were grown to an OD_600_ between 0.4 and 0.5 after which Isopropyl-β-d-1-thiogalactopyranoside (IPTG, Thermo Fisher Scientific, Waltham, MA, USA) was added at a final concentration of 0.5 mM to induce the expression. After 4 h, the culture was centrifuged at 4000 rpm for 10 min and the cell pellet was collected and resuspended in 100 mM HEPES buffer (1/10 of the final culture volume). To this, 50 µg/mL DNase I (Sigma) is added and sonicated at 60% amplitude for 30 s. The cell suspension was centrifuged again at the same conditions and the supernatant containing the protein was collected [25]. Proteins were purified by affinity chromatography using His-Pur Ni-NTA resin, and the purified protein was dialyzed against water overnight and lyophilized [26]

### 2.2. Protein Characterization

#### 2.2.1. Sodium Dodecyl Sulfate Polyacrylamide Gel Electrophoresis (SDS-PAGE)

Lyophilized protein was resuspended in phosphate buffer saline (PBS) for further analysis. SDS-PAGE was performed to check the size and purity of the protein. Each sample was heated with NuPAGE LDS sample buffer (Invitrogen, Waltham, MA, USA) at 95 °C before loading into precast Bis-tris 4–12% polyacrylamide gradient gel (Thermo Fisher, Waltham, MA, USA). The gel was stained with AcquaStain Protein Gel Stain (Bulldog Bio. Inc, Portsmouth, NH, USA) according to the manufacturer’s protocol and visualized under white light on a BioRad Chemidoc XRS imaging system.

#### 2.2.2. Mass Spectrometry (MS)

Mass spectrometry was performed on purified protein samples at the Core Synthesis and Analytical Services Facility (Center for Protease Research, North Dakota State University, Fargo, ND, USA). Purified protein was desalted through a desalting cartridge followed by LC-MS analysis for intact mass analysis on a Waters Synapt G2-Si HDMS (Waters Corporation, USA). UPLC was performed on an Acquity UPLC- I class with a Waters BEH C18 (2.1 mm × 100 mm) 1.7 µm column. A linear gradient of mobile phase A (0.1% formic acid in water) to B (0.1% formic acid in Acetonitrile) at a ratio of 90/10 (A/B) to 10/90 (A/B) was performed over 7 min and the column was maintained at 35 °C throughout the analyses. The total run time was 13 min. Later, 100 µL of desalted protein solution was mixed with 200 µL of 0.1% TFA in Water/Acetonitrile (50/50) and 10 µL of this mixture was injected at a rate of 0.5 mL/min. Mass spectrometric analysis was performed on a Waters Synapt G2-Si HDMS. The spectra were processed between 5000 to 50,000 Da at 1 Da/channel and analyzed using MaxEnt1 software (Waters Corporation, Milford, MA, USA) to obtain the protein mass.

### 2.3. Western Blot

The purified protein was then confirmed by semi-dry Western blot. Briefly, the protein was transferred to a nitrocellulose membrane on a semi-dry blotter (Owl^TM^ semi-dry electroblotting systems, Fisher Scientific, Waltham, MA, USA) at a constant voltage of 70 V for 60 min. After proteins were transferred to the nitrocellulose membrane, the membrane was blocked overnight at 4 °C using 5% non-fat milk as a blocking agent. The membrane was washed with Tris buffered saline plus Tween 20 (0.05%) and incubated with a 1:2500 dilution of an HRP-conjugated 6x-His Epitope Tag Polyclonal Antibody (Thermo Scientific, MA1-21315, Waltham, MA, USA) for one hour. The protein was detected by Western ECL substrate (Promega, Madison, WI, USA) according to manufacturer’s protocol and the bands were visualized using the Omega Lum^TM^ G gel imaging system (Aplegen Inc, Pleasanton, CA, USA).

#### 2.3.1. Susceptibility of the Thrombin Sensitive Peptide (TSP) to Spent Bacterial Media

The thrombin-sensitive peptide, HHHHHHDDDDKGF_D_PRGFPAGG (TSP), was custom synthesized by standard Fmoc synthesis (WatsonBio, Houston, TX, USA). The susceptibility of the peptide to the bacterial thrombin-like enzyme was assessed by exposing the peptide to media from an actively growing *S. aureus* culture [13]. Enzymatic activity of the media was initially determined by titrating culture exudate into a fluorogenic thrombin substrate, Boc-Val-Pro-Arg-MCA (Sigma Aldrich, St. Louis, MO, USA) [27]. TSP was then exposed to the media with varying enzymatic activity and incubated at 37 °C with shaking. The sample was analyzed with HPLC (mobile phase 0.1% TFA/water (A) 0.1% TFA/Acetonitrile (B), under a gradient flow of 5–90% at a flow rate of 1.0 mL/min) every 12 h through 48 h. Protein was detected at a wavelength of 215 nm. A similar experiment was conducted with the recombinant spider silk to check the susceptibility of the silk protein to the bacterial enzyme.

#### 2.3.2. Conjugation of Silk-TSP

TSP was chemically grafted onto the silk peptide using EDC-NHS chemistry [28,29,30]. Lyophilized silk was dissolved in a reaction buffer (i.e., MES at a pH of 6). It was then activated by adding 100 mM EDC [1-ethyl-3-(3-dimethylaminopropyl) carbodiimide hydrochloride] and 200 mM excess of NHS (*N*-hydroxy sulfosuccinimide) and allowed to react for 30 min at room temperature. The number of reactive carboxyl groups of silk was calculated, and TSP was added to get an equimolar ratio of carboxy groups in silk and amine groups of TSP. The peptides were allowed to react for 3 h at room temperature. The formed conjugate was then dialyzed to remove the unconjugated TSP. The conjugate was then analyzed by SDS-PAGE.

### 2.4. Differential Scanning Calorimetry Analysis to Determine the Formation of Conjugate

Thermograms of the reacting peptides were determined using nano DSC (TA Instruments, New Castle, DE, USA) and compared with the conjugate to confirm the formation of the conjugate. All the peptides were dissolved in PBS pH 7.5. Samples were degassed before loading into the nano DSC cells. The samples were scanned from 10 to 110 °C at a rate of 1 °C/min. A PBS-only scan was measured to check the background interference and deducted from sample scan during analysis. Nanoanalyze software was used to deconvolute and analyze the data.

### 2.5. Determination of CAC of the Silk Peptide and the Conjugate

The critical aggregation concentration (CAC) of silk was determined by the pyrene fluorescence method [31]. Briefly, 20 µL of 24 µg/mL (working concentration) of a pyrene solution was prepared in acetone and acetone was evaporated. Later, 980 µL of silk peptide in the phosphate buffer at different concentrations was then added to the pyrene, and the mixture was sonicated for 5 min and incubated for 1 h. The fluorescence spectra of the samples were recorded at an excitation of 336 nm and emission of 360–450 nm wavelengths. Intensity peaks at 379 nm (I1) and 393 nm (I3) were recorded. The ratio of I1/I3 was plotted against log concentration, and the point where a sharp inclination in the graph was noted as the CAC.

### 2.6. Preparation of Drug-Loaded Nanospheres

The model antibiotic vancomycin is available in the form of salt as vancomycin HCl commercially. To entrap the drug in the hydrophobic core of the silk nanosphere, the hydrophobic form of the drug, a vancomycin-free base, was prepared according to the previously published method [32]. Initially V-HCl was dissolved in water at a concentration of 70 mg/mL. Then, the pH was increased to 8.0 by adding 3N NaOH. The vancomycin-free base was then precipitated out and centrifuged at 3000 rpm for 10 min. The vancomycin-free base (V-FB) was then sequentially washed in 70% ethanol and methanol before finally solubilizing in PBS. This V-FB was also validated by HPLC (data not shown here). A simple dissolution method was used to prepare drug-loaded nanospheres at three different ratios of protein:drug [33,34]. The dissolution method ensures physical entrapment of the drug, which is preferred over its chemical conjugates. Briefly, during this method, the drug and the co-block polymer are individually dissolved in aqueous media followed by mixing of the solutions and sonication. In this specific experiment, a silk solution in phosphate buffer higher than CAC was added slowly to the V-FB drug solution in PBS and sonicated with a probe tip sonicator for 5 min followed by bath sonication for an hour. Sonication aids in the formation of nanospheres, driving the drug into the hydrophobic core of the nanosphere. The mixture was then incubated at room temperature for 1 h.

To calculate the encapsulation efficiency, nanospheres were centrifuged at 15,000 rpm for 1 h, and the supernatant containing the unencapsulated drug was collected and analyzed by a UV spectrophotometer (Spectramax m5, Molecular Devices, Downingtown, PA, USA) at a wavelength of 280 nm for the free drug. The unencapsulated drug was then subtracted from the total drug added to obtain the encapsulation efficiency.

### 2.7. Characterization of Nanospheres

The hydrodynamic size, polydispersity index (PDI), and zeta potential of formed nanospheres [35,36] were analyzed by Zetasizer Nano ZS 90 (Malvern Instruments, Malvern, UK). Morphological examination of the nanospheres was performed via transmission electron microscopy (TEM) at the core facility. A drop of the sample was placed on a 300-mesh formvar-carbon-coated TEM grid for 1 min. Phosphotungstic acid 0.1%, pH 7–8 was dropped onto the grid and allowed to stand for 2 min and then wicked off. After the grids were dried, images were obtained using TEM (JEOL JEM-2100; Peabody, MA, USA) running at 200 kV.

### 2.8. Antibacterial Activity of the Conjugated Nanospheres

Antibacterial activity of the nanospheres was tested by calculating the Minimum Inhibitory Concentration (MIC) using the microplate method and Kirby Bauer Zone of Inhibition [37,38]. Briefly, to determine the MIC, a culture of *Staphylococcus aureus* (ATCC 49230) was incubated overnight at 37 °C with shaking. The next day, the bacterial cell solution was diluted to 10^7^ CFU/mL and different concentrations (ranging from 1 ug/mL to 32 ug/mL) of either the vancomycin-free drug or vancomycin-loaded nanospheres were added to the bacterial solution. Two hundred microliters of this bacterial/vancomycin suspension were seeded in a 96-well plate followed by incubation at 37 °C for 24 h. The optical density of each well was recorded on the microplate reader (Epoch, BioTek, Winooski, VT, USA). Selected concentrations of the particles surrounding the determined MIC were taken and ZOI was conducted by the disc diffusion method. Briefly, 6 mm discs were placed in 96-well plate and 200 µL of drug-loaded particles were added to the wells and the discs were soaked for 24 h. After 24 h, the discs were dried and plated on S. *aureus* streaked on LB agar plates, and the cleared diameter was measured using digital calipers.

#### 2.8.1. In Vitro Infection Responsive Activity

In vitro drug release was determined using a dialysis method in which drug-loaded particles were incubated with bacterial growth media in a dialysis tube (molecular weight cut off 7 kDa) [39]. The bacterial growth media was obtained from an overnight culture of *Staphylococcus aureus* (ATCC 49230) or *Staphylococcus epidermidis* (ATCC 14990) at 10^7^ CFU/mL. The culture was centrifuged at 3000 rpm to separate the bacterial media from the cell pellet, and 1.5 mL of each bacterial media was incubated with nanospheres separately. The dialysis tube was placed in a beaker containing PBS as release media under stirring. The release media was collected at predetermined time points and analyzed by UV-vis spectrometer at 230 nm to determine the amount of released vancomycin from the nanospheres.

#### 2.8.2. In Vitro Drug Release Study

To assess the release profile of the formulations, in vitro drug release was performed by the dialysis method following the same protocol described above. Since the infection-responsive release study showed significant release of the drug only in the *S. aureus* media, the drug release profile was established using only this media. The formulation was incubated for 48 h with the bacterial media extracted from the *S. aureus* culture. The release media was collected every 3 h and analyzed for drug release by a UV–vis spectrometer. The amount of the drug release was plotted against time to create a drug release profile.

#### 2.8.3. In Vivo Septic Arthritis Model

An initial pilot study was done in a modified, induced septic arthritis rat model using a local inoculation of bacteria [40,41,42]. All studies were done under the supervision of the Institutional Animal Care and Use Committee (protocol A19068) at North Dakota State University. Sprague Dawley rats (*n* = 3/cohort) were inoculated with 20 µL of *S. aureus* culture (10^2^ CFU/mL diluted into sterile PBS) directly injected into the synovial space of the right knee joint of the rat under isoflurane anesthesia. Injections were done after shaving the joint and swabbing with betadine and isopropanol to sterilize the site. The infection was allowed to develop for 48 h during which time the animal was closely monitored, and pain was controlled with buprenorphine injections (0.03 mg/kg). A sterile buffer was injected into the other knee to act as a control. The development of disease was monitored by visible erythema and/or swelling of the joint along with hematological analysis of synovial fluid and histopathological analysis. After 48 h, rats were euthanized by isoflurane overdose and synovial fluid was collected from the joint space prior to the disarticulation and further processing of the limb. Synovial fluid was spread on blood agar plates (VWR, Radnor, PA, USA) [43] and incubated overnight at 37 °C. The number of colonies was counted the next day.

After establishing the septic arthritis disease model, treatment with either free drug, drug-loaded conjugate particles, or drug-loaded silk particles was given on day two after bacterial inoculation. Rats were divided into three treatment groups (Table 1). The first group received drug-loaded (14 mg/kg) conjugate nanoparticles in the infected knee locally delivered into the synovial joint space while the uninfected knee was injected with unloaded conjugate nanoparticles. The second group received drug-loaded plain silk SX_2_ particles into the infected knee while the uninfected knee was injected with unloaded SX_2_ particles. The third group received a free drug into the infected knee joint and sterile PBS into the uninfected knee. The treatment was continued for 48 h after which rats were euthanized and limbs were collected for histopathological analysis. Before euthanasia, synovial fluid was collected from the joint space of the infected knee and the control knee of all the groups and cultured on LB agar plates overnight. After incubation overnight, the number of colonies were counted.

## 3. Histology

Histopathology was performed on the infected and uninfected bones by hematoxylin and eosin (H and E) stain. Initially the bones were harvested from the rat after euthanasia and fixed in 10% neutral buffered formalin for 72 h. Bone was then decalcified by immersing in an EDTA solution (10% solution at pH 7.4) for 2 weeks and exchanging it every alternate day. After the bone was decalcified, it was embedded in paraffin wax and sectioned. These sections were then mounted on glass slides and stained with hematoxylin and eosin stains according to standard protocol. Clear Rite 3 was used to deparaffinize the sections and passed through several changes of decreasing gradient of alcohol and finally with water. The tissue section was then stained with hematoxylin, which stains the nuclei to a dark blue color followed by eosin, which colors the nonnuclear elements pink. After H&E staining, the tissue section was covered with a glass coverslip using a synthetic resin mounting media like cytoseal XYL. Stained slides were imaged at 40x using the MoticEasyScan Digital Slide Scanning microscope (Motic Digital Pathology, San Francisco, CA, USA).

## 4. Results

### 4.1. Protein Characterization

**Figure 2:**
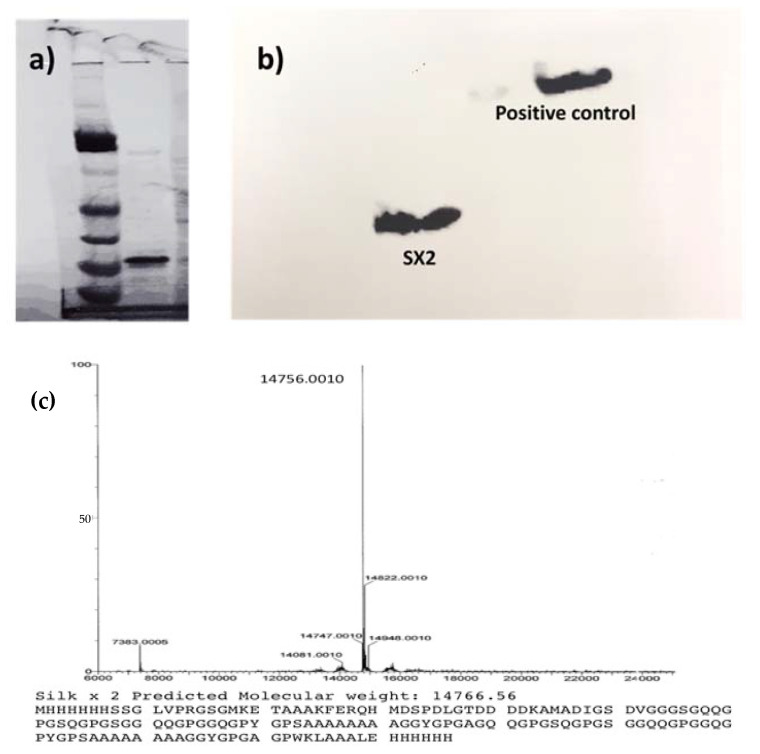
(**a**) SDS-PAGE of the purified protein showing the band at 15 kDa, (**b**) Western blot of the protein against an anti-his antibody confirms the production of the protein, (**c**) theoretical mass was predicted to be 14,766 Da while mass spectrometry of the silk protein was determined to be 14,756 Da.

Two repetitive sequence of the recombinant MaSp2 peptide (GGYGPGQQGPGGYGPGQQGPSGPGSAAAAAAAA) with a six histidine tag and an enterokinase site N terminal was cloned and expressed in *E. coli*. The recombinant protein was purified using Ni-NTA resin for His-Tag purification and validated by SDS-PAGE, Western blot, and mass spectrometry. The theoretical molecular weight of the protein was predicted to be 14,766 Da, which was confirmed by a band on the SDS-PAGE corresponding to 15 kDa (Figure 2a). The molecular weight was additionally confirmed by Electro Spray mass spectrometry after running through UPLC and showed a peak at a mass of 14,756 Da (Figure 2c). Finally, a Western blot was performed with an anti-His antibody, which further validated the production of the recombinant silk protein (Figure 2b).

### 4.2. Susceptibility of the Thrombin Sensitive Peptide (TSP) and Silk Protein to the Bacterial Media

**Figure 3:**
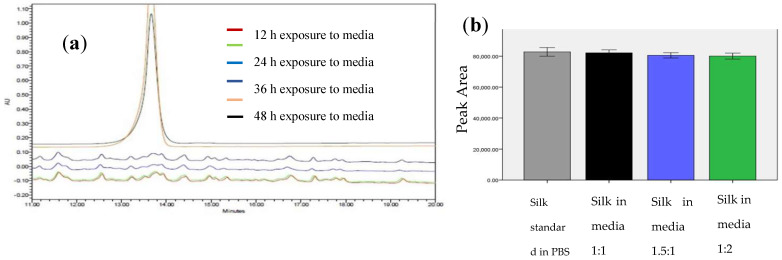
(**a**) HPLC chromatogram of TSP exposed to the bacterial media from *S. aureus* culture shows the disappearance of the peak at 13.5 min within 12 h of exposure to the media (bottom brown graph), while the peak was not only still evident after 48 h (black graph), but it was also relatively unchanged. (**b**) The area under the curve of the chromatogram of silk peptide exposed to the bacterial media, showing the stability of the peptide even after 48 h.

The susceptibility of the TSP to the bacterial thrombin-like enzyme was assessed by exposing the peptide to media from an actively growing *S. aureus* culture. Enzymatic activity of the bacterial culture was determined by a fluorogenic thrombin substrate, Boc-Val-Pro-Arg-MCA. TSP was initially titrated into media with varying enzymatic activity to determine the amount of enzyme required to cleave the peptide. The enzymatic activity was found to be 23 a.u. HPLC showed that TSP was cleaved within 12 h of exposure of the peptide to the bacterial media (Figure 3a), whereas the peptide was still intact upon exposure to water or even human thrombin at the same enzymatic activity (data not shown). Subsequently, susceptibility of the silk protein to the bacterial thrombolytic enzyme was analyzed in a similar manner and showed no significant difference in the degradation of silk even after 48 h at different concentrations of the media when compared to the control group without spent bacterial media (Figure 3b). This shows that TSP can be readily cleaved by the bacterial enzyme, whereas the silk protein is stable.

### 4.3. Creation of Silk-TSP Conjugate

**Figure 4:**
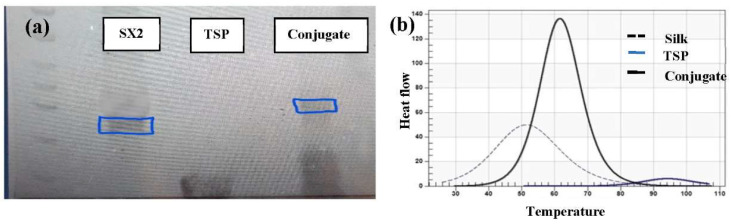
(**a**) SDS PAGE showing the molecular weight difference between silk and the conjugate, (**b**) DSC graph of silk, TSP and the conjugate showing increase in enthalpy of the conjugate.

Amide coupling by EDC/sulfo-NHS is one of the most commonly used strategies for cross-linking biomolecules. TSP was chemically grafted onto the silk peptide using EDC-NHS chemistry and confirmed by gel electrophoresis, which shows a shift in the band due to a slight increase in the molecular weight after conjugation (Figure 4a). It was also confirmed by DSC (Figure 4b). The thermogram of the conjugate is compared with the reacting peptides. The transition temperature Tm of the conjugate was found to be different from either of the reacting peptides, which confirms the presence of a new molecule, i.e., conjugate.

### 4.4. Characterization of Silk Nanospheres

**Figure 5:**
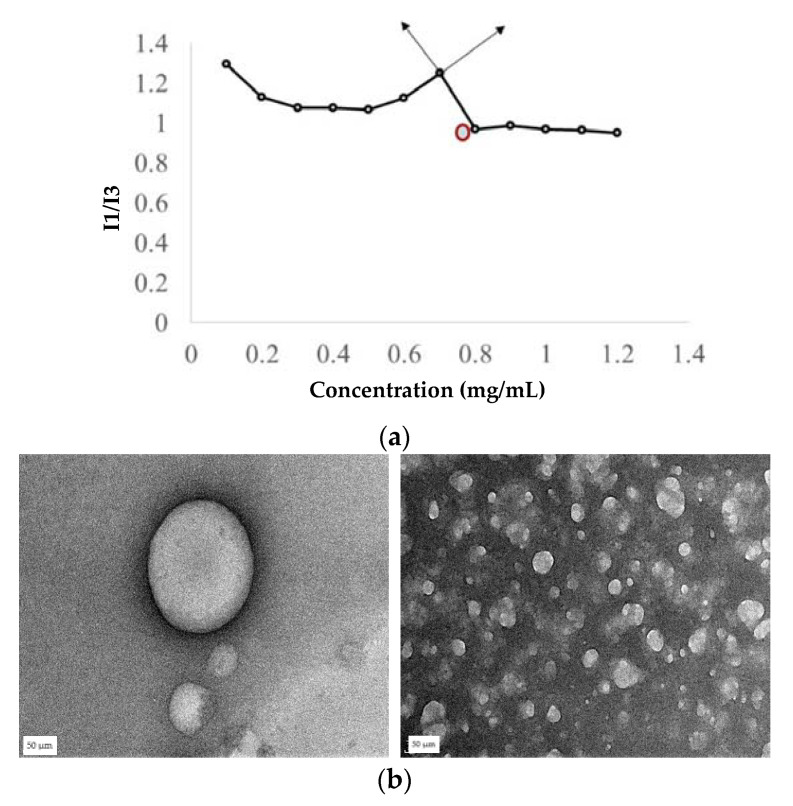
Critical aggregation concentration (CAC) of the conjugate was determined using (**a**) the standard pyrene fluorescence method. The concentration at the tangent on the graph, which shows a sudden fall in the intensity ratio, corresponds to the CAC (0.7 mg/mL). (**b**) TEM images of Vancomycin-loaded silk nanospheres show spherical nature of the particles.

Nanospheres are self-assembled structures containing a hydrophobic core to entrap a hydrophobic drug and hydrophilic shell for dispersion in the aqueous media [44]. Silk is an amphiphilic protein with a natural co-block polymer structure and an inherent tendency to form micelles [45]. The CAC of plain silk was determined to be 1.2 mg/mL (81 µM) by pyrene fluorescence while the CAC of the conjugate was found to be 53.7 µM. The vancomycin-free base was derived from vancomycin HCl, making the drug hydrophobic and allowing it to be incorporated into the inner core. The formed nanospheres were determined to be an average of 184 ± 12 nm in diameter with a Poly Dispersity Index (PDI) of 0.29 ± 0.12 and Zeta potential of −16 mV (Table 2). The size of the particles (less than 1 µm) is not anticipated to impact the delivery of the particles. Among different formulations, the first formulation containing drug:protein of 1:1 was found to have the highest encapsulation efficiency of 56% (Table 2). TEM of the conjugate micelles (Figure 5) confirmed the spherical architecture of the nanospheres.

### 4.5. Antibacterial Activity of the Conjugate Nanospheres

**Figure 6:**
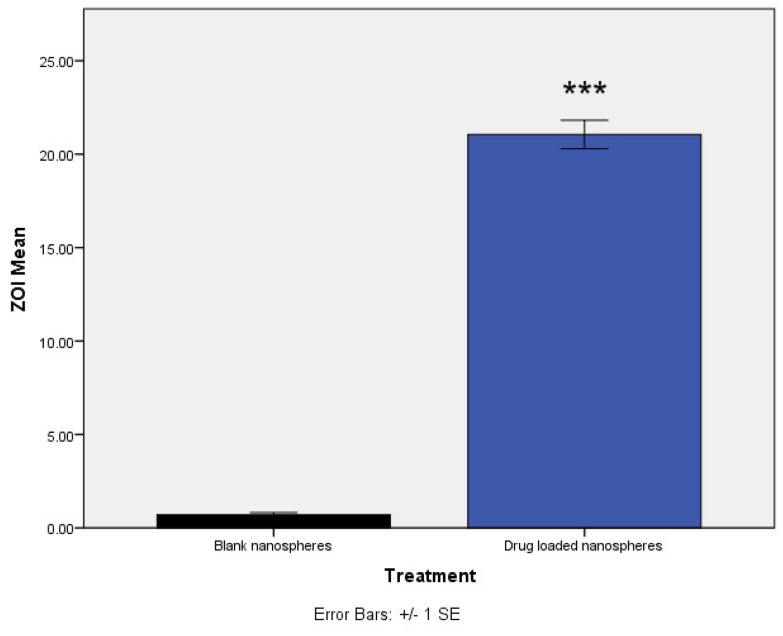
ZOI of blank and drug-loaded nanospheres at MIC (*** *p* < 0.001).

To explore the potential efficacy of this delivery system for the treatment of *S. aureus* septic arthritis, the antibacterial effect of Vancomycin-loaded nanospheres was first tested in vitro on *Staphylococcus aureus*. The Minimum Inhibitory Concentration (MIC) of the vancomycin-free drug was determined to be 2 µg/mL, while the MIC of the drug-loaded nanospheres was found to be 16 µg/mL based on their OD values. Once the MIC was established, antibacterial activity was tested by a Kirby Bauer Zone of Inhibition assay. ZOI of the nanospheres was found to be 19.1 mm (Figure 6, *n* = 3, *p* < 0.001) when compared to empty micelles, which is in accordance with the standard ZOI values of the free drug (>12 mm) for SA.

#### 4.5.1. In Vitro Infection Responsive Activity

**Figure 7:**
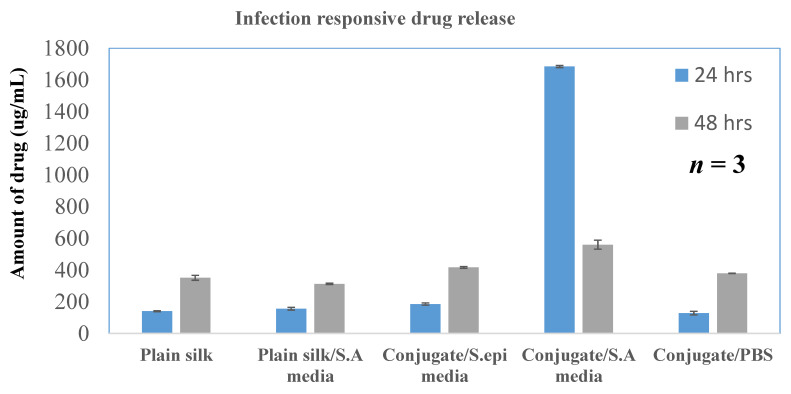
In vitro infection-responsive release of the drug-loaded particles against different media.

The release profile of the drug-loaded (2 mg) nanospheres under various conditions was recorded to evaluate infection responsive drug release. After 24 h of incubation, the cumulative release of the drug from the silk/TSP conjugate particles was recorded as 84.4% when exposed to the media from *Staphylococcus aureus*. The cumulative release from conjugate particles in the absence of media was found to be 18.9%. Plain silk particles showed a release of 15.6% and 17.5% when exposed to the media from SA and the buffer, respectively, indicating that although the silk particles do not show infection-responsive release, they do seem to be leaky. In order to confirm the infection-responsive release in the presence of SA specific enzymes, the release profile of conjugate particles was also determined in the presence of media from *S. epidermidis*. Drug release in response to *S. epidermidis* was found to be 20.8%, which is slightly greater than control particles (Figure 7).

#### 4.5.2. In Vitro Drug Release Study

**Figure 8:**
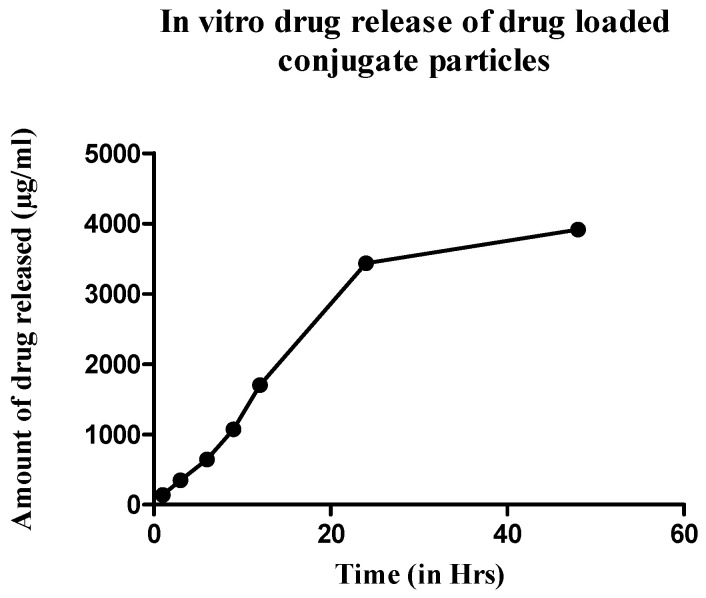
In vitro drug release profile of the drug-loaded conjugate nanospheres in the presence of bacterial media.

From the above study, it was determined that the formulation releases the drug only against infection media from *S. aureus*. To establish a drug release profile, the dialysis tubing method was used, and the sample was analyzed by UV spectroscopy. It showed that the maximum release of the drug (85%) was achieved within 12 h, with the release slowly declining over time, giving an almost bell-shaped curve. The cumulative drug release of the formulation is represented in Figure 8.

#### 4.5.3. In Vivo Drug Release in Septic Arthritis Model

**Figure 9:**
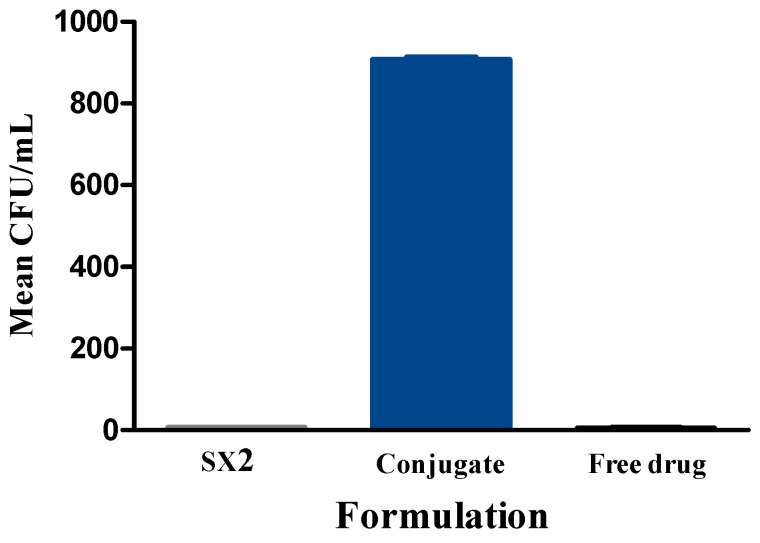
Colony count assay on synovial culture of the infected knee after treatment.

The disease model was established by inoculating the bacteria at a concentration of 10^2^ CFU/mL directly into the knee joint. After 48 h, synovial fluid was collected from both the infected and uninfected knee joints prior to collecting the joints. An overnight culture of the synovial fluid from the infected joint showed a lawn of bacteria while the uninfected knee did not grow any bacteria. The knees were also visually inspected for erythema and inflammation, which further confirms the development of septic arthritis. Treatment was given on day two. Bacterial culture from group one, the drug-loaded conjugate particles, showed an average of 40 CFU/mL, whereas group two, containing drug-loaded SX2 particles, showed 810 CFU/mL on an average (Figure 9). This confirms the in vivo infection-responsive release of the drug from the conjugate.

## 5. Histopathology

**Figure 10:**
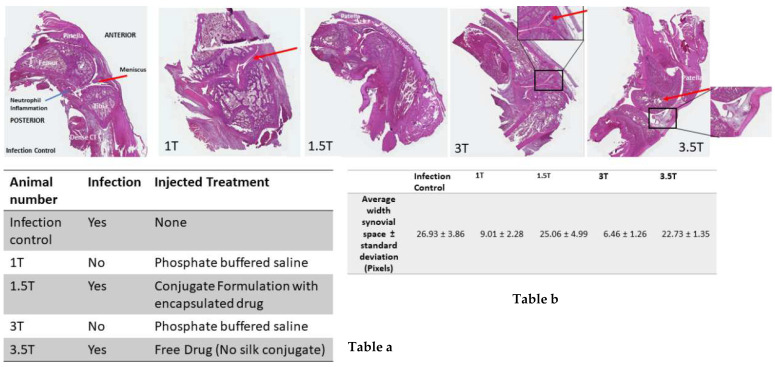
H & E staining images of bone samples from various treatment groups showing changes in the bone and synovium. Table a shows different treatment groups and their codes. Table b shows the width of the synovium quantified by image J in different groups.

In control animals injected with *S. aureus* without any treatment to establish that the current method was effective at establishing an infection, there was evidence of acute inflammation by marked neutrophilic infiltration in fibrotic synovium with thickened collagen septa (Dense CT). Histopathological results additionally showed higher levels of nucleated cell infiltration and some evidence of meniscus degeneration and shape change. Alternatively, in the control animals with no infection (1T and 3T), PBS was injected to assess the tissue response to injection. Hematoxylin and Eosin (H&E) staining showed narrow synovial lining with regular borders and normal joint structures (Figure 10). Furthermore, the triangular shape of the meniscus (red arrow in each image) is intact and unremarkable. In contrast, H&E staining of an infected joint injected with drug encapsulating conjugate particles (1.5T) showed a mild degeneration of the meniscus and vast vacuolar infiltration of the distal femur just superior to the epiphyseal growth plate (star). The synovium was also widened relative to 1T, indicating some inflammation to the knee joint space; however, it is unclear if this is due to the drug or the conjugate particles, since knees injected with the free drug only showed a similar, if not more inflamed, appearance (3.5T), with evidence of some meniscus degeneration and shape changes as well as signs of marked inflammation. The arrow on 3.5T indicates cellular infiltrates and vacuolar degeneration, whereas the star shows the sub patellar fat pad. Finally, the changes in width of the synovial space from multiple images were quantified using Image J and are reported as the average of all measurements with a standard deviation (Table b in Figure 10).

## 6. Discussion

The development of smart drug-delivery systems as an alternative to new drug discovery is essential to address the rising tide of resistance. Advances in drug delivery are lagging behind more traditional approaches to expand current drug classifications to address resistance; an emphasis should be placed on disease-responsive, local drug delivery systems. Disease-responsive elements might be integrated into characterized block copolymer biomimetics such as silk. Compared to systemic antibiotic administration, local drug delivery can decrease antibiotic dosing, avoid issues of patient compliance, and limit systemic side effect. Despite these advantages, clinical implementation and efficacy of many local antibiotic delivery systems (e.g., antibiotic loaded bone cement, etc.) is limited by a bolus jettison of drugs followed by sub-therapeutic antibiotic leaching [46]. A better strategy may lie in microenvironment-triggered local drug-release systems capable of providing disease- or tissue-targeted local drug delivery with the potential to overwhelm drug resistance mechanisms [9,47]. Tissue targeting, half-life, and pharmacokinetics are all aspects of controlled drug delivery systems that can be customized by modifying the drug delivery/encapsulating matrix. Molecularly manipulating bioinspired polymers that have defined structure–activity relationships (SARS) allows the design of smart biomaterials with embedded “protease switches” to act as an environmentally responsive drug delivery system. Spider silk mimicking MaSp2 peptides can be designed to include not only a balance of hydrophobic and hydrophilic amino acid blocks suitable for self-assembling nanosphere formation, but also a protease-responsive element in a single innovative biopolymer drug delivery system.

Creating the recombinant silk peptide with an integrated thrombin-sensitive element required (1) the chemical synthesis of a thrombin-sensitive peptide and (2) the expression and purification of a recombinant silk peptide. The thrombin-sensitive peptide was commercially synthesized using standard Fmoc synthesis and confirmed by HPLC (data not shown) and mass spectrometry. Concurrently, a recombinant MaSp2 peptide (GGYGPGQQGPGGYGPGQQGPSGPGSAAAAAAAA)_2_ with a six histidine tag and an enterokinase site N terminal to the silk-like peptide was cloned and expressed in *E. coli*. The susceptibility of the peptide to the bacterial thrombin-like enzyme was assessed by exposing the TSP/silk conjugate to media from an actively growing *S. aureus.* TSP was cleaved within 12 h of exposure to the bacterial media but was intact even after 24 h of exposure to the human thrombin or water controls and showed minimal degradation in human thrombin after 48 h (*n* = 3, Figure 3a). The proteolytic stability of the silk peptide still shows the presence of a stable peptide (Figure 3b), indicating that TSP was selectively cleaved by the enzyme. Subsequently, TSP was chemically grafted onto the silk peptide using EDC-NHS chemistry. The conjugate was analyzed initially by SDS-PAGE (Figure 4a), which showed a band slightly higher than the SX_2_. Since TSP is a very small peptide, gel electrophoresis is not precise in indicating the difference between conjugate and silk protein. Therefore, the conjugate was further analyzed by Differential Scanning Calorimetry (DSC) (Figure 4b). Notice that silk without TSP is shown in the dashed line, TSP alone is shown as a solid blue line (smallest peak), while the conjugate (solid black line) showed a shift in transition temperature in between that of silk and TSP and an increased intensity. After confirming that the conjugate was produced, the silk/TSP conjugate peptide was allowed to self-assemble into nanospheres. Unlike many other protease drug release systems that use a protease sensitive linker peptide to tether their drug payload to an insoluble matrix, spider silk peptides can be directly engineered to not only incorporate a protease-sensitive element but also to promote self-assembly via salting out [23,48] into micelles and microcapsules [22], allowing direct encapsulation of a bioactive drug. From the pyrene fluorescence method, the CAC for silk was determined to be 81 µM while the CAC of the conjugate was slightly less than the plain silk, 53.7 µM. Drug-loaded conjugate nanospheres were prepared by the dissolution method and the formed nanospheres were characterized by Zetasizer, which showed the average size of the particles to be 184 ± 12 nm with a negative zeta potential (Table 1) as expected for the amino acid sequence of Masp2. TEM of the conjugate nanospheres (Figure 5b) confirmed the spherical architecture of the nanospheres. TSP did not form nanospheres in the absence of silk, thus proving the importance of the silk protein in the formation of nanoparticles.

After forming the nanospheres, antibacterial activity of the formulation was evaluated by determining the MIC of the drug-loaded formulation and comparing it with the free drug. The MIC of the nanospheres was 16 µg/mL, which showed a ZOI of 19.1 mm, corresponding to standard ZOI values. Blank formulations did not show any ZOI suggesting that the drug is released from the encapsulated nanospheres to show antimicrobial activity (Figure 6). The infection-responsive release was assessed by incubating the formulations to different types of media. When exposed to the media from *S. aureus,* the conjugate showed a release of 85% and silk without TSP showed 15% (Figure 7), suggesting that TSP was cleaved by the bacterial enzyme to burst the nanospheres and release the drug, whereas plain silk was unable to rupture in the presence or absence of the enzyme. Furthermore, to confirm the specificity of the enzymatic release, drug-loaded formulations were also exposed to the media from *S. epidermidis*, which showed a release similar to PBS, suggesting that the vancomycin release was specific to the enzyme secreted from *S. aureus*. The drug release profile was established in the same manner of dialysis to assess whether the formulation showed an immediate release profile and the duration of release. It showed that drug release reached a maximum in 24 h (Figure 8) and started to decline throughout the remainder of the study. The release also corelates with the antibiotic release reported in the literature, which showed the maximum release of the antibiotic within 24 h of incubation [13]. This is again in correspondence with the initial HPLC evaluation of susceptibility of TSP, which showed that the TSP was cleaved within 12 h, although the formulation showed only 75% of release, potentially due to the saturation of the enzyme in the media.

Finally, to assess the efficacy of the infection-triggered drug-release system, the nanospheres were evaluated in a septic arthritis model. Septic arthritis is an inflammatory disease of the joints with an invasion of synovial membranes commonly caused by *Staphylococcus aureus* [42]. Typically, it is considered a secondary infection since the bacteria escapes from the bloodstream and localizes in one large joint such as the knee or hip but can also affect any other joint. Infection can spread to bones and different types of cartilage. The initial growth of bacteria is primarily in the synovial membrane, which results in a marked increase in leukocytes and neutrophils in synovial fluid. However, during later stages of the infection, the destruction of joint structures can be detected, particularly articular cartilage and subchondral bone loss. Systemic antibiotic therapy for 2–6 weeks [49] is the usual prescribed treatment for septic arthritis. In cases like septic arthritis, local delivery of the antibiotic appears to be a better approach than systemic delivery. Therefore, the septic arthritis model selected to test the local release of the drug from the formulations in the presence of infection was appropriate since it is a localized infection. Although systemic bacterial inoculation represents an accurate way to simulate disease development, it takes roughly 21 days for the disease to develop in animal models [41]. In order to hasten the disease development, septic arthritis was induced by local inoculation of the bacteria into the joint space. Septic arthritis was developed within 24 h of infection as confirmed by synovial culture and visual inspection of the knee joint.

After the treatment, drug-loaded conjugate particles showed significantly more bacterial clearing than drug-loaded plain silk SX2 particles. Some bacterial clearing was also observed with SX2, which suggests leakiness of the particles. Histological examinations on injected knee joints of the rat models in this present study were performed to verify injection sites as well as to provide evidence of inflammation from the induced septic arthritis by the injection of *Staphylococcus aureus*. Based on the current literature, a mild to moderate inflammatory reaction was expected at the injection site; however, the knee joint injection sites showed limited disruption in the skeletal muscle pattern and were difficult to visualize. Nevertheless, the remaining structures in the joint were evaluated with particular attention to the width of the synovial space and the shape of the meniscus. Hematoxylin and Eosin (H&E) staining showed a narrow synovial lining and the width of the synovial space was also quantified, which was slightly smaller than the infection control. Smaller synovial widths are generally associated with less inflammation. However, based on this pilot study, more analysis remains to be done on this measure. The analysis does indicate that an infection was established in these animals without evidence of clinical presentation noted in the animal during the experiment (i.e., no significant signs of pain, distress, or lameness).

## 7. Conclusions

Systemic administration of antibiotics is a common practice for wound infections and infections after hip or knee replacement surgeries. Apart from the long duration of treatment, systemic administration causes unnecessary toxicity, and poor penetration into the tissues may require higher doses. Lower concentrations at the disease site also augment the development of resistance by the bacteria. In such cases, localized delivery is more advantageous in terms of dosing as well as frequency of administration. Thus, we developed infection-responsive spider silk nanospheres, which can release the drug only at the site of infection. The use of spider silk as a drug carrier provides the advantage of encapsulating a large amount of the drug without leaching due to elastic and mechanical properties. Local delivery of these particles also ensures that the particles can release the required concentration of the drug at the disease site. This study also paves the way to develop other disease-specific drug release models by developing peptide linkers sensitive to specific enzymes related to the disease.

## Figures and Tables

**Table 1:** Cohort chart of animal groups.

Cohort	Bacteria	Formulation	Drug
0	Yes	No	No
1	Yes	Yes (conjugate)	Yes
2	Yes	Yes (SX2)	Yes
3	Yes	No	Yes

**Table 2:** Drug loading efficiency.

Protein: Drug (mg/mL)	EE (%)	Size (nm)	PDI
1:1	56	184 ± 12	0.3 ± 0.1
1:2	26.2	196 ± 4	0.4 ± 0.1
1:3	18.3	254.5 ± 13	0.4 ± 0.1

## Data Availability

The data presented in this study are available in “Infection Responsive Smart Delivery of Antibiotics Using Recombinant Spider Silk Nanospheres”.
